# A Novel Fault Diagnosis Method for Rotating Machinery Based on a Convolutional Neural Network

**DOI:** 10.3390/s18051429

**Published:** 2018-05-04

**Authors:** Sheng Guo, Tao Yang, Wei Gao, Chen Zhang

**Affiliations:** School of Energy and Power Engineering, Huazhong University of Science and Technology, Wuhan 430074, China; levykwok@hust.edu.cn (S.G.); gw@hust.edu.cn (W.G.); zhangchen710@yeah.net (C.Z.)

**Keywords:** convolutional neural network, fault diagnosis, vibration, wavelet transform

## Abstract

Fault diagnosis is critical to ensure the safety and reliable operation of rotating machinery. Most methods used in fault diagnosis of rotating machinery extract a few feature values from vibration signals for fault diagnosis, which is a dimensionality reduction from the original signal and may omit some important fault messages in the original signal. Thus, a novel diagnosis method is proposed involving the use of a convolutional neural network (CNN) to directly classify the continuous wavelet transform scalogram (CWTS), which is a time-frequency domain transform of the original signal and can contain most of the information of the vibration signals. In this method, CWTS is formed by discomposing vibration signals of rotating machinery in different scales using wavelet transform. Then the CNN is trained to diagnose faults, with CWTS as the input. A series of experiments is conducted on the rotor experiment platform using this method. The results indicate that the proposed method can diagnose the faults accurately. To verify the universality of this method, the trained CNN was also used to perform fault diagnosis for another piece of rotor equipment, and a good result was achieved.

## 1. Introduction

Large-scale rotating machines, such as steam turbines, wind turbines, and rolling mills, are ubiquitous in industries. With the development of technologies, the technical level and complexity of these systems are increased. Failure of these systems will lead to unexpected downtime, which will result in high operation and maintenance cost. Fault diagnosis, which aims to detect, isolate, and identify the fault before failure happens is, therefore, critical to ensure the safety and reliable operation of these systems.

Vibration signals are widely used for diagnosis of rotating machinery. There are many reported analysis methods, including wavelet transform, empirical mode decomposition (EMD) [1], Wigner-Ville distribution [2], Hilbert–Huang transform [3], order tracking [4], decision tree [5], rough sets theory [6], and principal component analysis (PCA) [7], etc. Among these methods, wavelet transform is a time-frequency domain analysis tool that provides better local characteristics of the signal. Due to this, it is often used in de-noising, feature extraction, and fault detection [8,9]. Wavelet transform was also integrated with other advanced algorithms, such as auto-associative neural networks [9], support vector machines [10], genetic algorithms [11], and support vector regression [12], among others, to enhance noise reduction, enable feature extraction, and facilitate multiple fault detection and classification.

With these successes, however, existing wavelet transform-based methods have some limitations. One is that they form the features extracted from wavelet transform coefficients in a one-dimensional vector, which is insufficient to describe the two-dimensional time-frequency domain wavelet transform and will result in information loss. The other is that feature selection and extraction significantly depends on expert knowledge, which is inflexible and difficult to obtain a generic solution.

To overcome these limitations, this paper proposes a novel fault diagnosis approach by integrating the continuous wavelet transform scalogram (CWTS) [13] with a convolutional neural network (CNN). In the proposed approach, wavelet transform decomposes vibration signals in different scales. The wavelet coefficients form the CWTS, which contain the complete time-frequency domain information of the vibration signals. Since the CNN has excellent multi-variable processing capabilities, it can take the full two-dimensional wavelet coefficients as input for fault diagnosis to achieve better performance.

Convolutional Neural Network is an emerging deep learning algorithm with reported successes in recognition of image [14], face [15], handwriting [16], action [17], materials [18], and speech processing [19]. For instance, in image recognition, the CNN takes original image as inputs and, therefore, avoids complex pre-processing. This is because the CNN has a special structure of local weight sharing. There are also some examples of CNN applications in disease diagnosis [20,21,22]. All of these applications show the advantages of CNNs in image and multivariate time series analysis, which indicates that CNNs have potential in diagnosis and prognosis [23]. However, through the inspection of these advantages, the applications of CNNs in fault diagnosis of mechanical equipment are very limited. A WDCNN (Deep Convolutional Neural Networks with Wide First-layer Kernels) method for fault diagnosis of a bearing is proposed in [24], but the influence of varying rotating speed on signals is not considered. This paper aims to introduce a new application of CNN in fault diagnosis. The contributions of the proposed approach are that:

1. For the first time, it integrates the CWTS and the CNN for fault diagnosis of rotating machinery. In this integration, the CNN has the multidimensional processing capability that can directly use two-dimensional CWTS as the input. This configuration takes full advantage of the CWTS and the CNN in a single deep learning framework;
2. The full two-dimensional wavelet coefficients are used in fault diagnosis without dimensionality reduction. The CWTS contains the complete time-frequency domain information of the vibration signals and avoids information loss of the original signal. Additionally, the wavelet transform also helps to remove noise from the raw signals at the same time;
3. A data preprocessing step is introduced to avoid the different distributions of the CWTS caused by different sample frequencies and different rotating speeds;
4. Parallel CNNs are used for fault classification in the experiment. Several CNNs are trained and each of them scores for a type of fault. Then the fault mode is obtained by comparing the scores of the CNNs.
5. The data pre-processing and the CNN algorithm are not data- and system-dependent. Thus indicates that the proposed solution is a universal, generic, and scalable one that can be applied to other diagnostic applications. Experiments on two different testbeds are presented to demonstrate the effectiveness and versatility of the proposed approach.

The paper is organized as follows: Section 2 elaborates the integration of the CNN and CWTS for fault diagnosis, with a detailed procedure of the proposed method; Experimental verification of the method is described in Section 3; Section 4 presents the experiments of the trained CNN on a similar experimental testbed, but with different configuration to verify the universality of the method; and, finally, concluding remarks are given in Section 5.

## 2. Proposed Method

As discussed above, the CWTS has been used in the fault diagnosis of rotating machinery. However, the existing methods only use the CWTS to extract features manually, which not only requires extensive knowledge of the system, but also results in information loss. Therefore, a CNN is introduced to process the CWTS with its great capabilities in image recognition. The integration of the CWTS with a CNN brings some immediate challenges, as follows:

1. The structure of the CNN and the format of the input image need to be defined. The structure of the CNN will influence the training time of the CNN. The format of the input images and the number of convolution layers have an influence on whether appropriate feature maps can be obtained.
2. The data format needs to be unified. Vibration data collected in different sample frequencies, with different rotating speeds, or from different equipment, will result in different distributions of the CWTS. This may cause difficulty in CNN recognition if the data format is not unified.

**Figure 1:**
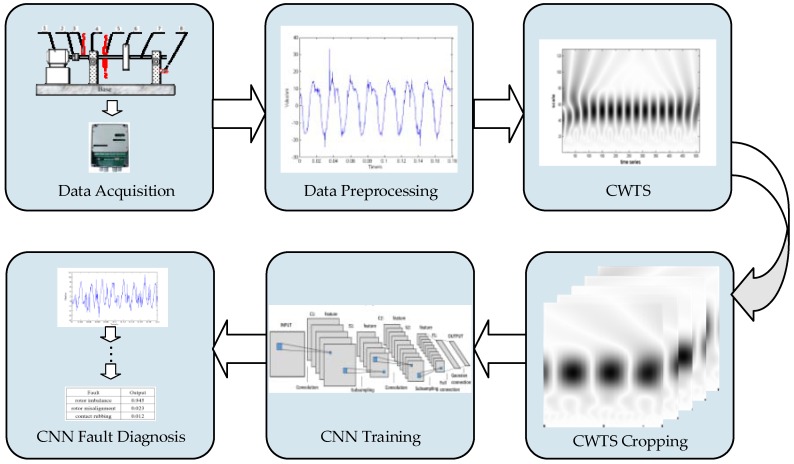
Flow chart of proposed fault diagnosis method.

The proposed approach aims to address these challenges in fault diagnosis of rotating machinery. Figure 1 illustrates the procedure of the proposed method, which consists of data acquisition (different types of fault data), data pre-processing (including data formatting), CWTS construction (decompose the vibration signal using the multi-scale continuous wavelet transform to obtain the CWTS), CWTS cropping (using part of the CWTS as the CNN input), CNN training, and real-time system diagnosis. Details of the each step of the proposed method are described below.

### 2.1. Data Acquisition

A rotating machinery can be operated with a variety of rotating speeds and loads. To perform fault diagnosis under various operating conditions, the vibration signals from the machine in a full speed range and a full load range need to be obtained for training. However, if the sample frequencies of the signals are not the same multiple of the rotating frequency, the different rotating speed will cause a substantial difference in CWTS. To eliminate this influence, vibration signal is collected (as a training instance) with the rotating speed information so that it will be taken into consideration when this instance is processed. Note that the rotating speed in a training instance is considered as constant as it is collected when the machinery is in a stable operating condition.

### 2.2. Data Preprocessing

First, the DC component of the vibration signal is removed as it does not contribute to fault diagnosis. The DC part is removed by simply subtracting the mean value of the signal. Note that if the vibration signal is from displacement sensors, the DC part will only denote the distance between the sensor and the rotating rotor. The DC part will also cause mistakes in the wavelet transform.

Second, the variation of rotating speed leads to changes in the CWTS. Since the rotating speed changes in operation when the operating mode changes, load changes, and during startup and shutdown, the CWTS will yield significantly different results if signals at different rotating speeds are not preprocessed. To eliminate the influence of rotating speed on CWTS, signal resampling with a virtual resampling frequency (VSF) is introduced. For the vibration signal in a training instance, as its rotating speed is known, the VSF is set as a frequency that is *q* multiples of the rotating speed. Note that *q* remains the same for all training instances. With this resampled vibration signal, every rotation of the rotor has the same number of sampling points. Then the wavelet coefficients corresponding to the same harmonic of the rotating frequency in different samples will locate at the same scale of CWTS.

Suppose a vibration signal x(k)(k=1, 2, …, m) is collected at a sampling frequency *f*(Hz) with *m* sampling data points. The rotating speed is *n* (rpm), corresponding to a machine rotating frequency $f_{m}=n/60$. Define *f_d_* as the virtual re-sampling frequency that is the required multiple number of times of the machine rotating frequency, i.e., $f_{d}={qf}_{m}$, where *q* is the required multiple number. To unify the sampling frequency as *f_d_*, the data is processed using the following method.

With re-sampling frequency *f_d_*, the *k*-th re-sampled data point should be $\bar{x}(k)=x(\frac{kf}{f_{d}})$. If *f* is a multiple of *f_d_*, then we only need to select $x(\frac{i\times f}{f_{d}}),(i=1,\;2,\;3,\;\cdots )$ as the new $\bar{x}(k)$. Otherwise, using a quartic polynomial interpolation function Φ with the original samples around $x(\lfloor \frac{kf}{f_{d}}\rfloor )$, the new $\bar{x}(k)$ (*k* = 1, 2, 3 …,) can be obtained by using Equation (1):

(1) $$\begin{array}{c} \bar{x}(k)=\Phi (\vec{K},\vec{X})\\ \vec{K}=\left(\left\lfloor \frac{kf}{f_{d}}\right\rfloor -1,\left\lfloor \frac{kf}{f_{d}}\right\rfloor ,\left\lfloor \frac{kf}{f_{d}}\right\rfloor +1,\left\lfloor \frac{kf}{f_{d}}\right\rfloor +2\right)\\ \vec{X}=\left(x\left(\left\lfloor \frac{kf}{f_{d}}\right\rfloor -1\right),\;x\left(\left\lfloor \frac{kf}{f_{d}}\right\rfloor \right),\;x\left(\left\lfloor \frac{kf}{f_{d}}\right\rfloor +1\right),\;x\left(\left\lfloor \frac{kf}{f_{d}}\right\rfloor +2\right)\right) \end{array}$$

After preprocessing, all data have the same length at the sampling frequencies that are the same multiples of the rotating frequency.

### 2.3. CWTS

The wavelet transform decomposes a signal in the time-frequency domain by using a family of wavelet functions. Different from Fourier transform, whose basis function is the sinusoidal function, wavelet transform uses the wavelet basis function, which is of finite bandwidth both in the time domain and the frequency domain. By scaling and translating the wavelet basis function, the signal can be decomposed with different resolutions at different time and frequency scales. The scaling and translation of a basic wavelet function can be mathematically described as:

(2) $$\Psi_{a,b}(t)={\left|a\right|}^{-\frac{1}{2}}\Psi (\frac{t-b}{a})\;a,b\in R\;a\neq 0$$

where Ψ*_a,b_*(*t*) is a continuous wavelet whose shape and displacement are determined by *a*, the scale parameter, and *b*, the translation parameter, respectively.

The continuous wavelet transform inherits and develops the localization idea of the short time Fourier transform (STFT). Different from STFT, scale and translation parameters *a* and *b* enable the adjustment of the resolution in time and frequency axes and, therefore, provide different frequency resolution and time resolution. The continuous wavelet transform is an ideal tool for signal time-frequency analysis and processing.

The continue wavelet transform of a signal *x*(*t*) is defined as the convolution of the signal *x*(*t*) with the wavelet function Ψ*_a,b_*(*t*). In this method, continuous wavelet transform is implemented to decompose the data from scale 1 to *l*, where *l* is usually equal to, or larger than, 2*q*:

(3) $$C_{a}(k)=\int x(t)\cdot {\bar{\Psi }}_{a,b}(t)dt$$

where *C_a_* (*a* = 1, 2, 3, …, *l*) is the wavelet coefficients of *x*(*t*) at the *a*-th scale and ${\bar{\Psi }}_{a,b}(t)$ is the complex conjugate of the wavelet function at scale *a* and translation *b*.

Continuous wavelet transform generates coefficients on different parts of the signal under different scaling factors. Using these wavelet coefficients, the signal in the time-frequency domain can be directly expressed by a two-dimensional image. The graph of the wavelet coefficients constructs the continuous wavelet transform scalogram (CWTS).

Putting all wavelet coefficients in a matrix ***P*** = [*C*_1_, *C*_2_, …, *C_l_*], it can be transformed to a gray matrix ***P***_new_ by:

(4) $$P_{\mathrm{new}}(i,j)=\left\lfloor \frac{P(i,j)-p_{\min}}{p_{\max}-p_{\min}}\times 255+\frac{1}{2}\right\rfloor$$

where *p*_min_ and *p*_max_ are the minimal and maximal elements of P, respectively. The value of element in ***P***_new_ represents a gray value in the range from 0 to 255. Therefore, ***P***_new_ is the continuous wavelet transform scalogram of the original signal.

**Figure 2:**
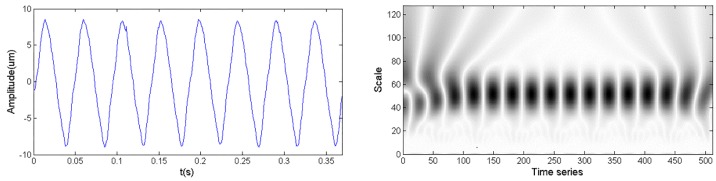
Time domain waveform and CWTS of a normal signal.

**Figure 3:**
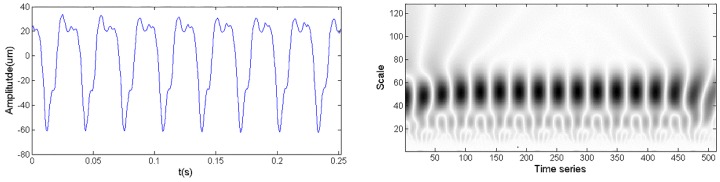
Time domain waveform and CWTS of a fault signal.

Figure 2 shows the time domain waveform and CWTS of a normal signal. As a comparison, Figure 3 shows the time domain waveform and CWTS of a fault signal with rotor imbalance. The signals both have 512 data points and are sampled at a frequency of 64*f_m_* and decomposed by the Morlet wavelet from a 1 to 128 scale. The horizontal axis represents the position along the direction of time signals, and the vertical axis represents the scale. The color of each point represents the magnitude of the wavelet coefficients.

As shown in Figure 2 and Figure 3, the CWTS of the fault signal is different from that of the normal signal. This result indicates the possibility to carry out fault diagnosis using CWTS. However, it is difficult to explicitly build a relationship between the CWTS and fault conditions. Although statistical feature [25] and one-dimensional vector were developed to recognize the difference, they are not sensitive to small changes in CWTS. For example, the wavelet grey moment (WGM) of the CWTS in Figure 2 and Figure 3 are 20.24 and 20.51, respectively. The difference between their WGM is trivial and is not reliable for diagnosis. In other words, it is difficult to detect rotor imbalance fault through WGM of CWTS obtained from the signals. To address this issue, CNN is proposed for fault diagnosis based on CWTS of vibration signals by taking full advantage of its capabilities in multidimensional signal processing and image recognition.

When choosing the wavelet type, we refer to the wavelet selection in other papers of machinery fault diagnosis. Zhang et al. [13] use eight types of wavelet to calculate the first-order WGM. WGM distributing lines of fault signals corresponding to eight wavelets are presented. It shows that three wavelets, Dmeyer, Meyer, and Morlet, have better distinguishability for machinery faults. Yan and Gao [26] use an energy-to-Shannon entropy measure to choose an appropriate wavelet for a vibration signal. The test signal extracted by the Morlet wavelet has the higher energy-to-Shannon entropy ratio than the other wavelet types listed in the paper. It shows that the Morlet wavelet is the most appropriate wavelet for analyzing the signal. According to the analysis in these papers, the Morlet wavelet was chosen as the wavelet used in this paper. If other wavelet functions commonly used in vibration signal analysis are selected, this method may also have a good result.

### 2.4. CWTS Cropping

CWTS obtained from continuous wavelet transform usually has a large number of pixels. Recognition of large images often requires a more complex CNN structure and more computation, which lead to longer training and computing time. On the other hand, large images will diminish the effects of small local features and reduce the sensitivity and accuracy of fault diagnosis. To accommodate this, CWTS cropping is introduced, which is conducted with the following three principles:

1. The cropping result must contain at least the continuous wavelet transform coefficients of one complete rotating period.
2. The length of one side of the square result must be greater than 2*q*.
3. If the coordinate of the pixel at scale axis *i_a_* is greater than the coordinate at the time axis *i_b_* or the coordinate to the last point of the sample *m* − *i_b_*(*i_a_* > *i_b_ or i_a_* > *m* − *i_b_*), then the pixel cannot be used as the output.

The first principle is to ensure that the result contains the complete information of one period. The second principle is to obtain the wavelet transform of low scales from 1 to 2*q*, which often have the characteristics of the fault. Oil whirl, for instance, has fault characteristics in scales from *q* to 2*q* of CWTS. The third principle is introduced to avoid the following scenario: when the center of wavelet transform window is located in the first or last several points of the sample, there will not be enough points to perform the wavelet transform when the scale parameter is larger.

Meanwhile, as the fundamental rotating frequency *f_m_* is the major and common constituent of the vibration data, it corresponds to a considerable fraction of area in CWTS. If the signal is not synchronized, the difference in CWTS caused by fundamental rotating frequency *f_m_* will be significant and affect the accuracy of diagnosis.

Following these three principles and the needs of signal synchronism, a CWTS cropping scheme is proposed. First, *P*_0_, the phase of fundamental rotating frequency *f_m_*, is calculated by Fourier transform for every samples. Next, the first point after 2*q*, which has a zero phase of one multiple of the rotating frequency, is chosen as the start coordinate of cropping in the time axis. Thus, the start coordinate *i_c_* can be calculated by:

(5) $$i_{c}=\left\lfloor 3q-\frac{P_{0}}{360}\times q+\frac{1}{2}\right\rfloor \;(0\leq P_{0}<360)$$

Finally, the output can be obtained by extracting 1 to 2*q* in the scale axis and *i_c_* to *i_c_* + 2*q* − 1 in the time axis from the original scalogram.

**Figure 4:**
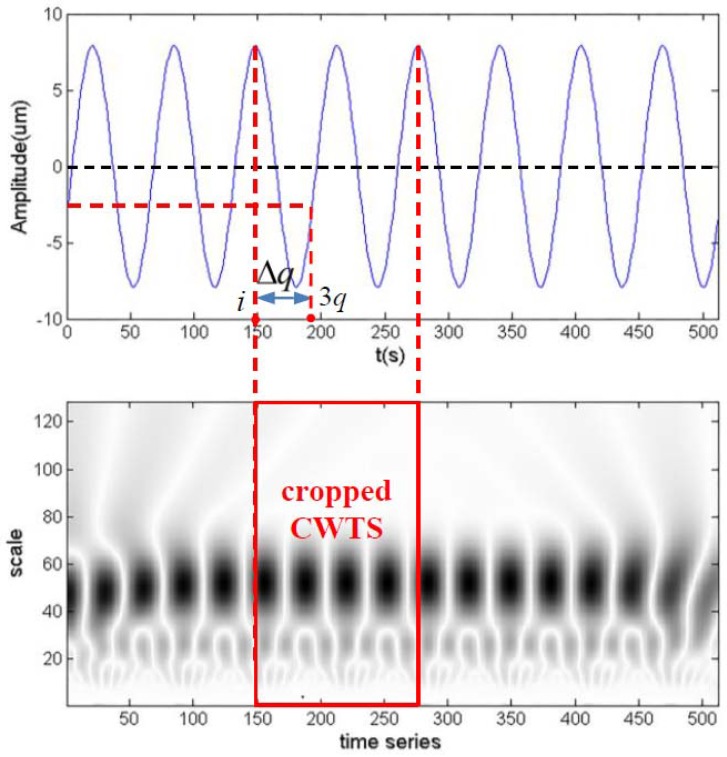
CWTS cropping of the signal in Figure 2, above: fundamental rotating frequency waveform of the signal, below: the CWTS of the signal.

Figure 4 illustrates the CWTS cropping process of the signal in Figure 2. *P*_0_ of the signal is 250.6, *q* is 64, and the time series number corresponding to *P*_0_ $\Delta q=\lfloor \frac{P_{0}}{360}\times q+\frac{1}{2}\rfloor =45$. Thus, 1 to 128 in the scale axis and 147 to 274 in the time axis index that is cropped as the output.

Using the above method, the influence of different starting phase of the one1 multiple of the rotating frequency can be eliminated. In addition, it helps to improve the speed of convergence compared to the cropping method without considering the one multiple of rotating frequency. After this step, we obtain a number of square preprocessed CWTSs as the training input of the CNN.

### 2.5. CNN Training

A convolutional neural network (CNN) is a kind of neural network that uses a convolution operation to replace the general multiplication in a neural network. It has excellent performance in dealing with data with a grid structure. Convolution operations improve the machine learning system through three important concepts: sparse interaction, parameter sharing, and equivariant representation [27]. Sparse interaction is achieved by making the size of convolution kernels much smaller than the size of the input. It reduces the computational complexity of algorithm and improves its statistical efficiency. Parameter sharing refers to using the same parameters in multiple functions of a model. The parameters of each convolution kernel are the same when dealing with different positions of the input. Equivariant representation roots in the properties of convolution operation, which is equivariant to any translation functions. This means that the features can be acquired no matter where they are located in the input [28].

**Figure 5:**
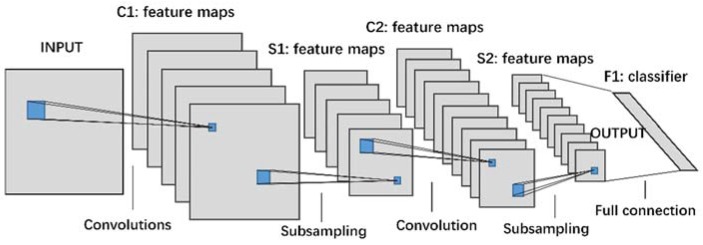
Structure of a CNN.

CNN has many different structures. The basic structure of CNN used in this paper, Figure 5, consists of two types of layers, feature extraction layer (also known as convolution layer) and feature mapping layer (or pooling layer) [14]. Each computing layer of the CNN, such as C1, S1, C2, and S2 in Figure 5, is composed of a number of feature maps. Each feature map is mapped to a plane, and the convolution operations share the same convolutional kernel at different locations of the feature map. The feature mapping structure uses the sigmoid function as the activation function.

The convolution layer consists of a number of feature maps. Each neuron of the convolution layer receives a limited range of the input feature maps and performs the convolution operation on the input. For each input feature map, *K* output maps will be obtained if the convolution layer has *K* convolution kernels. Suppose the input *X* is the matrix of *M* × *N*, the output of the convolution layer can be computed as:

(6) $$h_{i,j}^{k}=\theta ({\left(W^{k}*X\right)}_{i,j}+b_{k})$$

where $h_{i,j}^{k}$ is the value at coordinate (*i, j*) of the convolution layer’s output of the *k*-th feature map by the *k*th convolution kernel, *i* = 1, 2, …, *M* − *s* + 1, *j* = 1, 2, …, *N* − *s* + 1, *W^k^* ∈ *R^s^*is a weight vector representing the *k*-th filter, s is the kernel size, *b_k_* is the bias of the *k*-th feature kernel, and *θ*(*x*) is the activation function, which is set as the sigmoid function in this paper.

Each convolution layer is followed by a pooling layer to conduct aggregate statistics on characteristics at different location of the feature map. This will reduce the dimension of convolution features of a convolution layer by pooling. Two types of pooling, average pooling and maximum pooling, are widely used. The average pooling is employed in this research, which is computed as:

(7) $$p_{i,j}=\frac{1}{s^{2}}\sum_{m,n=1}^{s}h_{(i-1)\times s+m,(j-1)\times s+n}$$

where $P_{i,j}$ is the value at coordinate (*i, j*) of the pooling layer’s output, *s* is the pooling size, $h_{\left(i-1\right)\times s+m,\left(j-1\right)\times s+n}$ is the value at corresponding place of the convolution layer’s output.

A classifier is then trained for fault diagnosis. In this paper, a fully-connected neural network is used as a classifier. The input of the neural network is a one-dimensional vector constructed by all the values in feature maps. The fully-connected neural network calculates the dot product between the input vector and the weight vector, plus a bias. The outcome is sent to the sigmoid function in the output layer for diagnosis.

To fully determine the CNN structure, some parameters need to be determined. Such parameters include the number of convolution layer, the number of convolution kernels in each layer, the size of kernels, the pooling size of each layer, the learning rate of the neural network, and the format of the training output.

1. Number of convolution layers and number of convolution kernels: The number of convolution layers depends on the size of the input CWTS image. More global characteristics of the image requires a higher number of layers and convolution kernels. However, the convergence speed will decrease with the increase of convolution layers or convolution kernels.
2. Size of kernels and pooling size: To reduce the training time and increase the convergence speed, a small kernel size and pooling size is often used. It also requires that the input and output images of each layer must have integer pixels.
3. Learning rate of the neural network: A high learning rate may lead to divergence of training. On the contrary, a low learning rate will lead to slow convergence. In general, the learning rate needs to be determined in training by trial-and-error to ensure both the stability and learning speed of training.
4. The input and output format of the fully-connected neural network: The input of the neural network is a one-dimensional vector formed by all the values in the feature maps. An *n* × 1 zero vector is created with n being the number of fault modes. If the *k*-th fault mode is detected, the *k*-th value of the output vector is set as 1 while all other values are 0.

With all initial parameters, the CNN is trained for fault diagnosis with a supervised learning algorithm. The basic idea of training is to adjust the weights and bias of the CNN by minimizing the residual. First, the residual of the fully-connected layer is calculated by a squared error loss function. Then error back propagation is carried out from the last layer to the first layer using the chain rule. The pooling layer uses the upsample to propagate errors back. For an average pooling layer, errors will be equally distributed in the pooling area. The convolution layer uses deconvolution for error back propagation. Deconvolution is performed by performing convolution with the reversed convolution kernel. After obtaining the errors of each layer, a gradient descent method is applied to update the kernels, weights, and bias of the convolution layer and the fully-connected layer in the direction of steepest descent.

**Figure 6:**
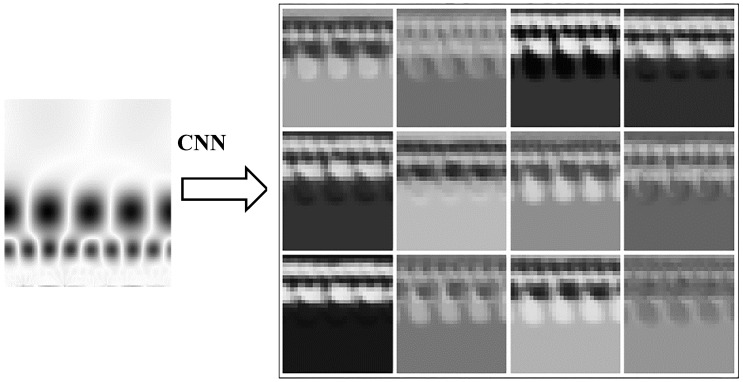
CNN output feature maps of a rotor misalignment fault signal CWTS, left: the CWTS of a rotor misalignment fault signal, right: twelve 29 × 29 feature maps obtained by the CNN.

Figure 6 (left) shows a 128 × 128 CWTS of a rotor misalignment fault signal. By using the CNN with a structure given in Figure 5, the original CWTS generates twelve 29 × 29 feature maps as shown in Figure 6 (right). This shows that the feature maps concentrating on different parts of CWTS are obtained by the CNN. Then the fully-connected neural network can classify the fault accurately.

### 2.6. CNN Fault Diagnosis

To perform fault diagnosis using the trained CNN, the raw vibration data are transformed to the same format of the training data. The transformed data are decomposed with the continuous wavelet transform to obtain the CWTSs. The CWTSs are then cropped to construct the input of the CNN. The CNN output is the result of fault detection, which indicates the detected fault mode.

## 3. Experiment Analysis

### 3.1. Fault Data Acquisition

**Figure 7:**
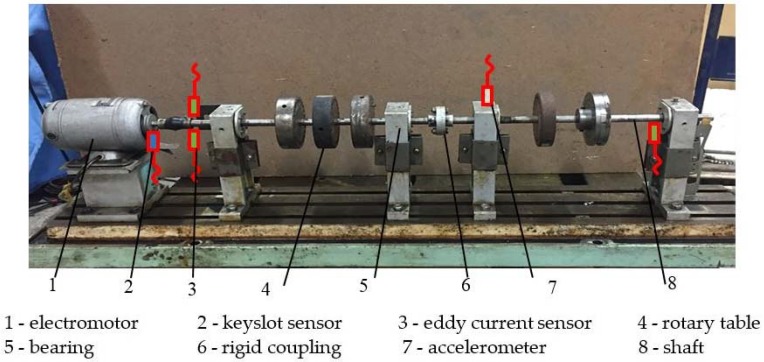
Structure of the rotor testbed.

Figure 7 shows the rotor testbed [13] that is used in this experiment. Different fault modes can be easily injected in the testbed. In this research, four fault modes, including rotor imbalance, rotor misalignment, bearing block looseness, and contact rubbing, are injected to verify the proposed method. The sampling frequency is selected as 64 times that of the rotating frequency. As discussed in the previous section, the sampling frequency must be an integer multiple of the rotating frequency. The data are then separated into samples with the same length. Each sample contains 512 data points, which equals eight rotation periods of the rotor. To cover the full working rotating speed range and operating conditions, the data at the machine’s stable operation, startup, and shutdown phases are all collected for each fault mode and healthy system (before the fault mode is injected). Note that since two sets of sensors are used, the normal healthy system will have two cases. One is for the displacement sensor and the other is for the acceleration sensor. That is, the total operating conditions in this study has six cases, rotor imbalance/misalignment/healthy from the displacement sensor and bearing block looseness/contact rubbing/healthy from the acceleration sensor.

Different types of sensors are used for different fault modes as each sensor is sensitive to a certain type of fault model. Displacement sensors are used for the diagnosis of rotor imbalance and misalignment, whereas acceleration sensors are used for the diagnosis of bearing block looseness and contact rubbing. For each type of fault, we carried out three or four experiments. In these experiments, the fault location or fault degree was changed. Taking unbalanced fault as an example, we tried to fit screws with different weights on different rotary tables. A total of 120 samples are randomly selected for each fault mode. The data covers the full rotating speed range, including machine startup and shutdown phases. The same amount of samples under normal healthy conditions are also obtained. Among the 120 samples for each fault mode and healthy system, 60 samples are randomly selected for training while the remaining 60 samples are used for testing.

### 3.2. Data Processing

**Figure 8:**
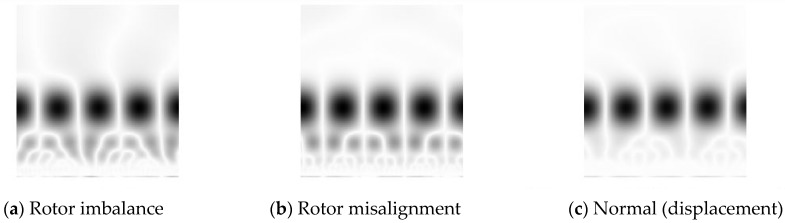
Preprocessed CWTSs of the displacement data.

**Figure 9:**
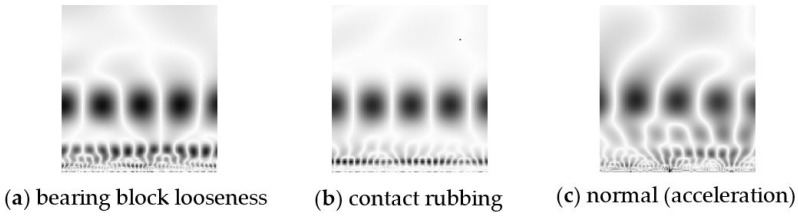
Preprocessed CWTSs of the acceleration data.

As discussed above, the data are sampled at a frequency that is the same multiple of the rotating frequency. The DC component of the data is then removed. The Morlet wavelet is used to obtain the CWTS for each sample (containing 512 data points). In this experiment, the Morlet wavelet is used because it has a similar shape feature with fault signals. Each sample is decomposed over a scale from 1 to 128. Thus, the CWTS of each sample has a resolution of 128 × 512 pixels. In CWTS cropping, 128 × 128 pixel pictures are obtained from the original scalograms using the proposed cropping method. A 128 × 128 pixel picture is used in this research because it represents two complete rotating periods and facilitates the CNN design in the next step. Figure 8 and Figure 9 show the cropped CWTSs of different fault modes.

Using the concept of parallel neural networks, four CNNs are trained in parallel for the above-mentioned four fault modes in which each fault mode corresponds to a CNN. Table 1 lists the input and output of the four CNNs.

These four CNNs are with the same structure, which contains four convolution layers and four pooling layers, 20 5 × 5 kernels in the first convolution layer, 30 5 × 5 kernels in the second convolution layer, 50 4 × 4 kernels in the third and fourth convolution layer, and the pooling size of the four pooling layers is 2 × 2. The training of CNN is set as 6000 iterations to guarantee convergence and accuracy. The learning rate of neural network is 0.005 in the first 3000 iterations to allow for rapid convergence and changes to 0.001 in the remaining iterations to ensure fine tuning for accuracy improvement. The selection of these parameters is problem dependent and obtained by trial and error. In this procedure, some other samples that do not belong to training or test samples are used as validation samples. When adjusting parameters, we try to make the training and validation samples obtain good classification results at the same time.

MATLAB is used to implement the training on the computer with an i7-4790K CPU, GTX750Ti GPU, 8 GB memory, and a 1 TB hard-drive [29]. Four CNNs are trained at the same time with GPU calculation. It takes about 13 h to guarantee convergence of the four CNNs.

### 3.3. Experimental Results

To verify the proposed approach, the remaining 360 testing samples are tested with the trained CNNs. Each sample is sent to two CNNs for diagnosis. It takes 2 s to process the test. Therefore, the proposed approach can be used for real-time automatic diagnosis. The outputs of the CNNs with the same sensor type are compared. A fault threshold $F_{d}$ and a no-fault threshold $H_{d}$ are given for fault detection. If the output of one CNN (fault mode F1) $y_{1}$ is greater than the fault threshold ($y_{1}>F_{d}$), while the output of the other CNN (fault mode F2) $y_{2}$ is smaller than the no-fault threshold ($y_{2}<H_{d}$), the fault corresponding to the first CNN F1 is detected. If the output of one CNN (fault mode F1) $y_{1}$ is between the no-fault threshold and the fault threshold ($H_{d}<y_{1}<H_{d}$), it is considered that the presence of fault mode F1 is uncertain and needs more data to analyze. In this experiment the fault threshold $F_{d}$ is set as 0.6 and the no-fault threshold $H_{d}$ is 0.4. Table 2 and Table 3 summarize the diagnosis result and accuracy, in which accuracy is defined as the correctly-detected number divided by the total test numbers. Table 2 and Table 3 show that the accuracies for all four fault modes are greater than 88%, which indicates that the proposed approach is effective in fault diagnosis of rotating machinery.

For a comparison study, the proposed approach is compared with the existing method [13] in which the first-order wavelet gray moment (WGM) is used for diagnosis. Table 4 shows the results from the WGM method. Another method using wavelet gray moment vector is compared with this method [30]. The method extracts first order wavelet gray moment vector (WGMV) from continuous wavelet transform coefficients of the signals. Then a probabilistic neural network (PNN) is used for fault classification. The WGMVs are also classified by SVM. The results of the method using the same training and test data are listed in Table 5.

Compared with Table 2 and Table 3, the results show that the method proposed in this paper has better diagnosis accuracies in all fault modes than WGM, WGMV-PNN, and WGMV-SVM methods. The CWTS contains more fault information than WGM and WGMV. The CNN can be used as a perfect feature exaction method of the CWTS through training. Table 4 shows that the WGM method cannot detect rotor imbalance. The lower performance of the WGM method is mainly due to the energy proportion of fundamental and higher harmonics of the rotating frequency, which has substantial influence on the gray level distribution of CWTS.

If the displacement signals of contact rubbing or bearing block looseness are presented to displacement CNNs or the acceleration signals of rotor imbalance and misalignment are presented to displacement CNNs, there will not be a misclassification. We choose the displacement signals collected at the same time as the contact rubbing and bearing block looseness samples. The signals are presented to displacement CNNs after data processing. Additionally, the acceleration data of rotor imbalance and misalignment are presented to acceleration CNNs. Then the output of the CNNs are compared with the fault threshold $F_{d}$ to obtain the diagnosis results. For each fault, 60 samples are chosen. The results presented in Table 6 and Table 7 shows that all the misclassification rates are less than 10%. The misclassification rate of all 240 samples is 5.42%. This shows that misclassification will not happen with this method.

### 3.4. Misclassification Analysis

Table 2 and Table 3 show that the proposed method does not correctly classify some test samples. This section provides some in-depth analysis to pinpoint the root cause of misclassification. There are two main types of incorrect outputs. Type 1 is that the outputs of two CNNs are both greater than the fault threshold ($y_{1}>F_{d}$ and $y_{2}>F_{d}$ at the same time), while type 2 is that the corresponding element is slightly smaller than the threshold ($H_{d}<y_{1}<F_{d}$ and $y_{2}<H_{d}$ at the same time).

**Figure 10:**
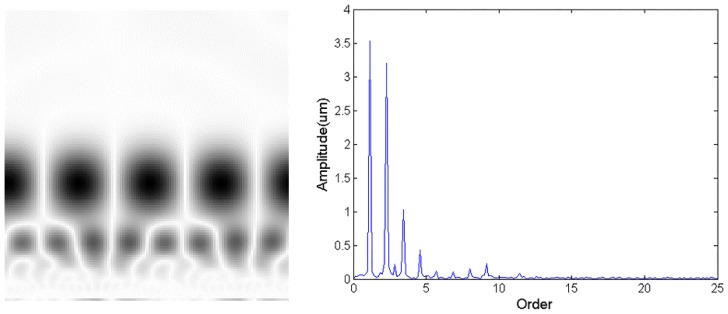
CWTS and spectrum of the imbalance test data.

One incorrectly classified sample in rotor imbalance falls into a type 1 misclassification. Figure 10 shows the CWTS and spectrum of the data in this sample. Its output for the rotor imbalance CNN and misalignment CNN are 0.79469 and 0.85982, respectively. Both of them are greater than the fault threshold of 0.6. The analysis of this sample reveals that the amplitude at the second harmonic of the rotating frequency is high, which indicates that it is possible that rotor misalignment and rotor imbalance occur at the same time.

**Figure 11:**
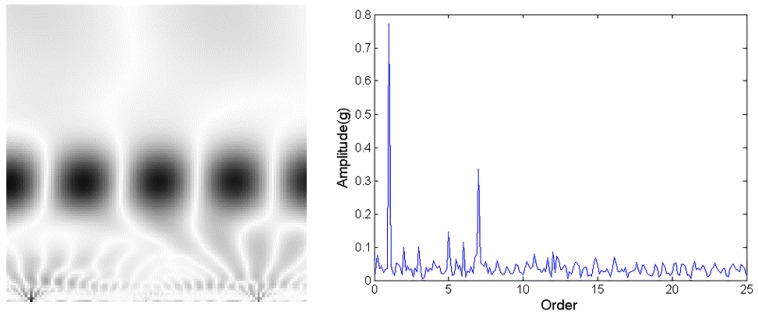
CWTS and spectrum of the contact rubbing test data.

For the type 2 misclassification, one misclassified sample data is from contact rubbing. The classified outputs by contact rubbing CNN and bearing block looseness CNN are 0.47452 and 0.01160, respectively. The output of contact rubbing CNN is less than the fault threshold of 0.6. Figure 11 shows that the high gray level points at the lower portion of the picture are not obvious, and the amplitude of higher harmonics in spectrum are not very high. Contact rubbing may be slight. This problem can be solved by including more training data.

## 4. Universality of CNN Fault Diagnosis

**Figure 12:**
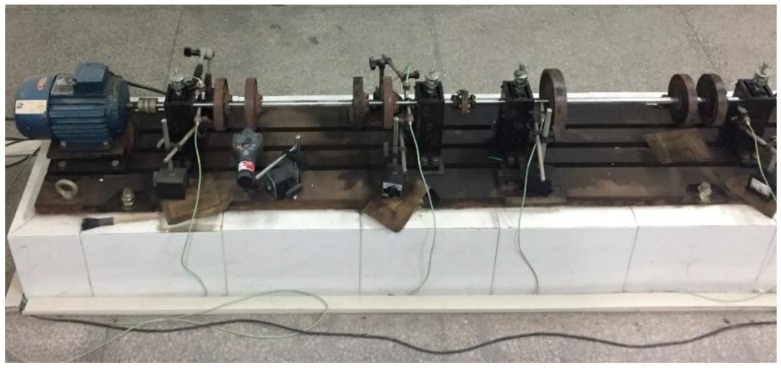
Structure of the gas turbine rotor testbed.

The objective of this research is to propose an accurate, robust, and universal solution for rotating machinery fault diagnosis. While the accuracy and robustness are demonstrated in Section 3, more research is needed to show the proposed method is universal, which indicates that the trained CNN from one equipment can be extended to the diagnosis of other equipment with similar structure or similar function. Universality is critical for most intelligent fault diagnosis algorithms as one with a high level of universality will greatly reduce the design and maintenance costs. To verify the universality of the proposed CNN fault diagnosis method, the trained CNN is applied to fault diagnosis of a gas turbine rotor testbed as shown in Figure 12. This testbed has a similar structure with the rotor testbed, but with a longer and thicker shaft and bearings of different sizes.

The vibration data is collected and processed in the same manner as that for the rotor testbed, as discussed in Section 3. The displacement sensing data of two faults, i.e., rotor imbalance and rotor misalignment, are used in this case study. Table 8 shows the diagnosis results with the same CNNs trained in Section 3 based on rotor testbed data. It is clear from Table 8 that the diagnosis results of all faults are greater than 70%, which demonstrates that the proposed approach is a universal and generic solution for diagnosis of other rotating machines. Note that this offers fast deployment of fault diagnosis with existing trained CNNs. With more data from the new equipment, the CNNs can be trained and updated to further improve the performance.

## 5. Conclusions

This paper proposes an accurate, robust, and universal deep learning-based fault diagnosis method for rotating machinery. The proposed approach is built upon the continuous wavelet transform and convolutional neural network. The novelty is that the CWTS constructed from the continuous wavelet transform that contains the complete two-dimensional wavelet coefficients is directly used in a CNN-based fault diagnosis without dimensionality reduction to avoid information loss. A series of experiments on a rotor testbed with four different fault modes are presented to demonstrate the effectiveness of the proposed approach and compared with the existing methods. To demonstrate the universality of the proposed approach, the trained CNNs are applied to fault diagnosis of a different testbed with similar, but different, structure. The results demonstrate that the proposed approach is a universal and generic solution.

## Figures and Tables

**Table 1:** Input and output of the CNNs.

Fault Mode	Sensor Type	Input/Count	Output	
Rotor imbalance	Displacement	rotor imbalance/60 rotor misalignment/60 normal (displacement)/60	1—rotor imbalance	0—others
Rotor misalignment			1—rotor misalignment	0—others
Contact rubbing	Acceleration	contact rubbing/60 bearing block looseness/60 normal (acceleration)/60	1—contact rubbing	0—others
Bearing block looseness			1—bearing block looseness	0—others

**Table 2:** Diagnosis result and correct rate using displacement CNNs.

Fault Mode	Test Samples	Correct Number	Accuracy
Rotor imbalance	60	55	91.67%
Rotor misalignment	60	60	100.00%
Normal (displacement)	60	59	98.33%

**Table 3:** Diagnosis result and correct rate using acceleration CNNs.

Fault Mode	Test Samples	Correct Number	Accuracy
Contact rubbing	60	56	93.33%
Bearing block looseness	60	53	88.33%
Normal (acceleration)	60	56	93.33%

**Table 4:** Diagnosis results of the WGM method using the same data.

Fault Mode	Test Samples	Correct Number	Accuracy
Rotor imbalance	60	0	0%
Rotor misalignment	60	53	88.33%
Contact rubbing	60	19	31.67%
Bearing block looseness	60	17	28.33%

**Table 5:** Diagnosis results of the WGMV-PNN and WGMV-SVM method using the same data.

Fault Mode	Test Samples	WGMV-PNN Correct Number	WGMV-SVM Correct Number	WGMV-PNN Accuracy	WGMV-SVM Accuracy
Rotor imbalance	60	51	52	85.00%	86.67%
Rotor misalignment	60	50	52	83.33%	86.67%
Contact rubbing	60	54	53	90.00%	88.33%
Bearing block looseness	60	49	48	81.67%	80.00%

**Table 6:** Misclassification numbers and rates when displacement data of contact rubbing and bearing block looseness are presented for displacement CNNs.

Fault Mode	Misclassification Number of Rotor Imbalance CNN	Misclassification Number of Rotor Misalignment CNN	Misclassification Rate
Contact rubbing	1	0	1.67%
Bearing block looseness	2	3	8.33%

**Table 7:** Misclassification numbers and rates when acceleration data of rotor imbalance and rotor misalignment are presented for acceleration CNNs.

Fault Mode	Misclassification Number of Contact Rubbing CNN	Misclassification Number of Bearing Block Looseness CNN	Misclassification Rate
Rotor imbalance	4	0	6.67%
Rotor misalignment	2	1	5.00%

**Table 8:** Diagnosis results of the gas turbine rotor testbed using the same CNNs.

Fault Mode	Test Samples	Correct Number	Accuracy
Rotor imbalance	60	43	71.67%
Rotor misalignment	60	52	86.67%
Normal (acceleration)	60	59	98.33%
